# People with serious mental illness are at higher risk for acute care hospitalization in Israel, 2000–2019

**DOI:** 10.1186/s13584-022-00544-7

**Published:** 2022-09-08

**Authors:** Ethel-Sherry Gordon, Rinat Yoffe, Nehama Frimit Goldberger, Jill Meron, Ziona Haklai

**Affiliations:** Health Information Division, Ministry of Health, Jerusalem, Israel

**Keywords:** Serious mental illness, Psychiatric case register Israel, Acute care hospitalizations, Psychiatric bed reduction, Standardized discharge ratio

## Abstract

**Background:**

People with severe mental disorders have higher mortality rates and more chronic physical conditions than the general population. Recent reforms in the Israeli mental health system included reducing the number of psychiatric hospital beds (“Structural Reform”), establishing community- based rehabilitation services (“Rehabilitation Reform”), and the transfer of governmental responsibility to the Health Maintenance Organizations (HMOs) (“Insurance Reform”). We examined how these changes have impacted the physical health of people with severe mental illness as reflected in acute care hospitalizations.

**Methods:**

Data from the National Psychiatric Case Register were linked with data from the National Hospital Discharges Database for 2000–2019. Acute care discharges from public hospitals were identified for people who had a psychiatric hospitalization with a diagnosis of severe mental illness (SMI, ICD-10 codes F10-F69 or F90-F99) within the preceding 5 years. The discharge rate of SMI patients was compared to that of the total population by age, diagnosis group, and period of hospitalization. Total and age-standardized discharge ratios (SDR) were calculated, using indirect standardization.

**Results:**

The SDR for total acute care hospitalizations showed that discharge rates in 2016–2019 were 2.7 times higher for the SMI population than expected from the total population. The highest SDR was for external causes (5.7), followed by respiratory diseases (4.4), infectious diseases (3.9), skin diseases (3.7) and diabetes (3.3). The lowest SDR was for cancer (1.6). The total discharge rate ratio was lowest at ages 65–74 (2.2) and highest at ages 45–54 (3.2). The SDR was lowest for females at ages 25–34 (2.1) and for males at ages 18–24 (2.3). SDRs increased over the study period for all diagnoses. This increasing trend slowed at the end of the period, and between 2012–2015 and 2016–2019 there was a small decrease for skin and liver diseases, the SDR was stable for cancer and the increase was smaller for respiratory, infectious and circulatory diseases and diabetes.

**Conclusion:**

This study showed higher hospitalization rates in people with SMI compared to the total population. These differences increased between 2000 and 2019 following the opening of alternative services in the community, possibly due to a higher likelihood of psychiatric hospitalization only for those with more severe mental disease. We recommend that general practitioners and mental health professionals in the community be made aware of the essential importance of good physical healthcare, and collaborate on health promotion and disease prevention in the SMI population.

**Supplementary Information:**

The online version contains supplementary material available at 10.1186/s13584-022-00544-7.

## Background

Excess mortality in people suffering from serious mental illness (SMI) has been known for decades, and is well documented and reviewed [1–5]. In recent years, excess mortality in people with schizophrenia and with bipolar disease have been among the OECD quality indicators for mental health [6], with international comparisons reported in their Health at a Glance series [7]. The average OECD rate ratio of mortality in those with mental illness, compared to the total population, was 4.0 for schizophrenia and 2.9 for bipolar disease, for 11 reporting countries in 2015–2017.

The poor physical health and high prevalence of chronic physical disease of people with SMI has also been widely reviewed and discussed, such as by Osborn [8] and Scott et al. [9]. The high metabolic syndrome for people with schizophrenia was reported in a review article by De Hert et al. [10], and their physical health problems were reviewed by von Hauswolff-Juhlin et al. [11]. Robson et al. [12], and De Hert et al. [13], discuss their diseases and the causes for their increased morbidity, including lifestyle factors such as high smoking rates and alcohol consumption, low levels of physical activity, poor diet contributing to high obesity levels, and the deleterious effects of medication for mental conditions.

Phelan et al. [14] and Maj [15] have called for improvements in the physical care of SMI patients, and De Hert [16] has recommended detailed treatment guidelines. One recurring issue is to specify the heath care professional responsible for monitoring their physical health—is it their primary physicians, or their mental health physicians and nurses?

In Israel, a detailed study of excess mortality among people with a recorded psychiatric hospitalization was reported in 2011 [17], and updated data were published in the annual statistical abstracts of Mental Health [18] and by the OECD [6, 7]. Chronic physical conditions and the use of health services by people with mental disorders were studied by Levinson et al. [19] using data from the Israel National Health Survey. They concluded that after controlling for sociodemographic and risk factors, people reporting mood, anxiety and substance abuse disorders were at significantly higher risk for chronic pain, respiratory conditions, cardiovascular conditions, diabetes or any chronic physical condition compared to those without these mental disabilities. In a recently published study of the largest health provider in Israel, people with a recorded schizophrenia diagnosis were found to have a higher mortality risk, increased risk for most chronic physical conditions, higher emergency room and hospitalization risks and longer mean hospitalization duration than the rest of the population [20].

In Israel, three major reforms in the mental health system have occurred in the last two decades. Following a national policy of de-hospitalization and advancing community-based treatment [21], the number of beds was reduced by half between 1994 and 2006, the largest decrease occurring between 2005 and 2006, of a third of the beds in 2005 (“Structural Reform”). In 2000, the passage of the Community-Based Rehabilitation of the Mentally Disabled Act led to the development of a full rehabilitation system for people with mental disabilities (“Rehabilitation Reform”) [18], and in July 2015 the responsibility for the provision of psychiatric services was transferred from the Ministry of Health to the health maintenance organizations (HMOs) (“Insurance Reform”) [22]. These changes, in particular the last one, which united physical and mental health provision under the same umbrella, should provide for improvement in the physical and mental health care of those with mental disabilities.

To help assess the burden of physical disease in Israel among people with SMI, we undertook this study of their acute care hospitalizations and discharge diagnoses from all public hospitals, between 2000 and 2019. Acute care hospitalizations of those with SMI have not been studied widely in Israel. Similar studies have been conducted in England for those with schizophrenia, schizoaffective disorder, and bipolar disease [23] and for those with personality disorders [24].

## Methods

Data were merged, by encrypted identification number, from two national Ministry of Health databases: the National Psychiatric Case Register (NPCR), which records all admissions and discharges to psychiatric inpatient facilities in Israel, and the National Hospital Discharges Database (NHDD), which records all acute care hospitalizations in Israel, with demographic information, hospitalization departments and patient diagnoses.

The SMI population was defined as all people, aged 18–74, in the NPCR who ever had a discharge diagnosis of F10–F69 or F90–F99 (ICD-10). For each year between 2000 and 2019 we identified people from this population who had a psychiatric inpatient hospitalization that year or within the prior 5 years. We compared their acute care discharge rates from public hospitals in the NHDD for all non-maternity hospitalizations at ages 18–74 with those of the total population. We used indirect age adjustment to calculate the standardized discharge ratio (SDR) of the two groups by computing the ratio of actual discharges in the SMI group to the expected number based on the total population rate. The adjusted rate for the SMI group was then calculated from the total population rate and this ratio.

Age groups used for standardization were: 18–24, 25–34, 35–44, 45–54, 55–64 and 65–74 Age specific rate ratios (RRs) were also calculated. The analysis was done for five 4-year periods: 2000–2003, 2004–2007, 2008–2011, 2012–2015 and 2016–2019, for the total number of discharges as well as discharges with specific groups of diagnoses. The analysis was repeated, stratified by sex, and a further analysis was done for maternity hospitalizations only.

In order to better assess the morbidity of people with SMI, we used the ICD-9-CM code of the primary and secondary diagnoses as recorded in the NHDD database to define the diagnostic groups. Hence, each discharge could be included in several groups, thus giving recognition to chronic diseases, such as diabetes, which may not have been the primary diagnosis of hospitalization.

We also compared the SDRs for urgent hospitalizations, defined as those admitted through the emergency room, with those for planned hospitalizations in public hospitals and with those for planned hospitalizations in private hospitals. Private hospitals have no emergency rooms, and typically perform elective procedures and operations.

Confidentiality was strictly assured since the authors who analyzed the data had no access to the identity of the people linked by the encrypted administrative databases.

## Results

Between 1995 and 2019, 122 thousand people, were hospitalized at least once in psychiatric facilities, forming the SMI population as defined above. Males accounted for 57% of the SMI group. Details of psychiatric hospitalization data can be found in the yearly statistical abstracts published by the Ministry of Health [18].

Between 2000 and 2019 there were 11.5 million non-maternity discharges from acute care departments in public general hospitals of people aged 18–74, of which 201 thousand were in the SMI group with a psychiatric hospitalization in the previous five years. Additionally, there were 3 million maternity discharges of which 6803 were in the SMI group.

**Fig. 1:**
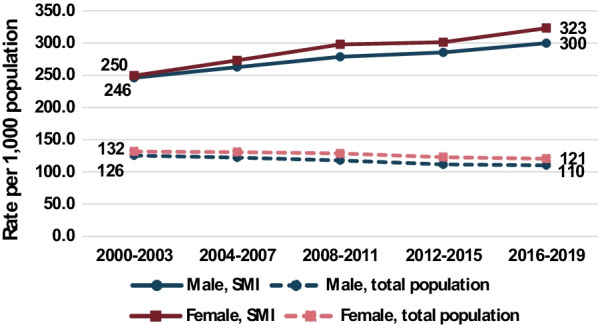
Age-standardized rates of all acute care discharges from public hospitals, 2000–2019. Rate per 1000 population, for people with SMI and total population, aged 18–74

Figure 1 shows the age-adjusted discharge rates for males and females by period for the two groups. The discharge rate increased in both sexes for the SMI group, among females by 30%, from 250 per 1000 in 2000–2003 to 323 in 2016–2019, and among males by 22%, from 246 to 300. In contrast, the corresponding total population discharge rates decreased by 8% among females and 12% among males.

The SDRs of the SMI group compared with the total population by sex and diagnoses are shown in Table 1 for the most recent period, 2016–2019. Total discharges were 2.6 times higher for the SMI group than predicted by the total population. The highest SDR, 5.7, was found for external causes, which includes suicide and self-inflicted injury with the very high SDR of 51.2. The highest disease group SDR was respiratory diseases (4.4), followed by infectious diseases (3.9), skin diseases (3.7) and diabetes (3.3). Other diseases, urinary, circulatory, digestive system and musculoskeletal, had SDRs between 2.8 and 2.6. The lowest SDR was for cancer, 1.6. The SDRs for females were higher than for males for most diseases.

**Table 1:** Standardized discharge ratios (SDR) for SMI group compared to total population, for acute care discharges from all public hospitals in 2016–2019, aged 18–74, by sex and diagnoses (based on any diagnosis in hospital record)

Diagnosis group (ICD-9-CM codes)	Standardized discharge ratios (SDRs)			Total number discharges	
	Total	Male	Female	All population	Thereof: SMI group
Infectious diseases (001–139)	3.9	4.0	3.9	144,421	4051
Cancer (140–208)	1.6	1.7	1.7	309,847	3556
Diabetes (250)	3.3	3.0	3.8	437,098	10,026
Circulatory (390–459)	2.7	2.4	3.0	879,583	16,401
Respiratory (460–519)	4.4	4.1	4.7	353,037	10,930
Digestive system diseases (520–579)	2.7	2.5	3.0	420,552	8260
Urinary diseases (580–599)	2.8	2.4	3.4	308,869	6040
Skin diseases (680–709)	3.7	3.5	3.8	115,439	3028
Musculoskeletal diseases (710–739)	2.6	2.4	2.8	242,929	4453
External (E800–E999)	5.7	4.7	7.1	259,296	10,567

All non-maternity discharges	2.6	2.7	2.7	2,525,192	47,701
**Specific diagnoses**					
Septicemia (038)	5.2	5.1	5.1	13,166	467
Pneumonia and influenza (480–488)	5.3	5.1	5.4	66,161	2448
Liver disease (571–573)	4.1	3.8	4.4	57,222	1683
Chronic renal failure (584)	4.9	4.4	5.3	49,679	1648
Acute renal failure (585)	2.7	2.4	3.3	106,658	1963
Suicide and self-inflicted injury (E950–E959)	51.2	42.6	64.7	9588	3546
**Maternity discharges**			**0.4**	**699,726**	**1331**

All SDRs were significant with *p* <  = 0.001

Among specific diseases, particularly high SDRs were found for septicemia (5.2), flu and pneumonia (5.3) and liver disease (4.1), while chronic renal disease had a much higher SDR (4.9) than acute renal disease (2.7). Suicide and self-inflicted injury had an extremely high SDR (51.2) and it was even higher (64.7) for females.

In contrast to discharges for external causes and for diseases, maternity discharges were lower for the SMI group than predicted by the total population, SDR = 0.4.

**Fig. 2:**
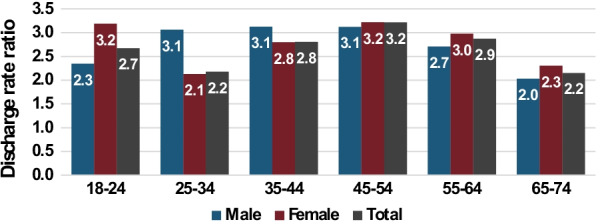
Rate ratios of all acute care discharges from public hospitals, by age and sex, 2016–2019. People with SMI compared to total population, aged 18–74

Figure 2 shows the RRs for non-maternity discharges in the SMI group compared to the total population, by age and sex for 2016–2019. The total RR is highest for ages 45–54 and lowest for ages 25–34 and 65–74, 3.2 compared to 2.2.

Although for ages 18–24 the RR was higher for females than for males (3.2 compared to 2.3) it was considerably lower for females aged 25–34 (2.1 compared to 3.1) lower at ages 35–44 (2.8 compared to 3.1) and similar for ages 45–54.

RRs were lowest for males aged 65–74 (2.0) and for females aged 25–34 (2.1).

**Fig. 3:**
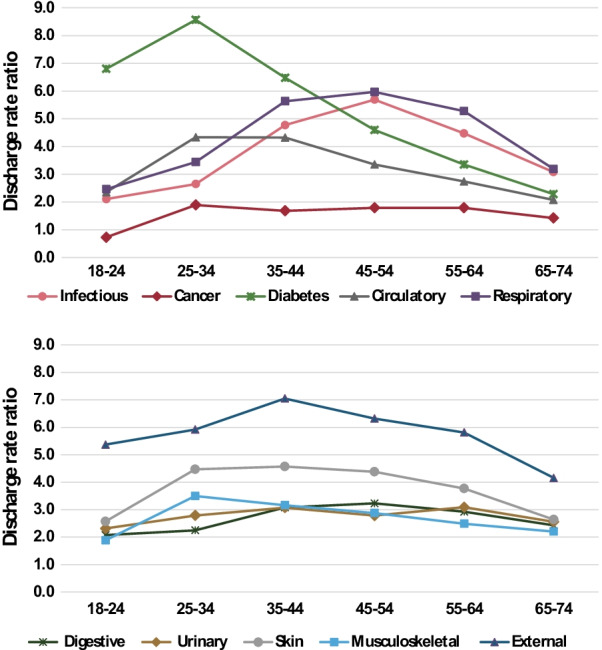
Rate ratios of all acute care discharges from public hospitals, by age and diagnoses, 2016–2019. People with SMI compared to total population, aged 18–74

Figure 3 shows the RRs in 2016–2019 for the SMI group compared to the total population by age and diagnosis group. All RRs were lower at ages 18–24, increased to higher values between ages 25–54 and were lower at ages 65–74. In general, the ranking of diagnosis groups found in Table 1 was maintained at all ages, with highest RRs for external and respiratory diagnoses and lowest for cancer. An exception was diabetes which had the highest RRs at ages 18–24 and 25–34, but with RRs decreasing rapidly with age, and among the lowest at ages 55–64 and 65–74. Interestingly, infectious diseases had relatively low RRs at ages 18-24 and 65–74, but high at 45–54.

Details of the RRs for Figs. 2 and 3, with 95% confidence intervals (CIs), are given in Additional file 1: Table S1.

**Fig. 4:**
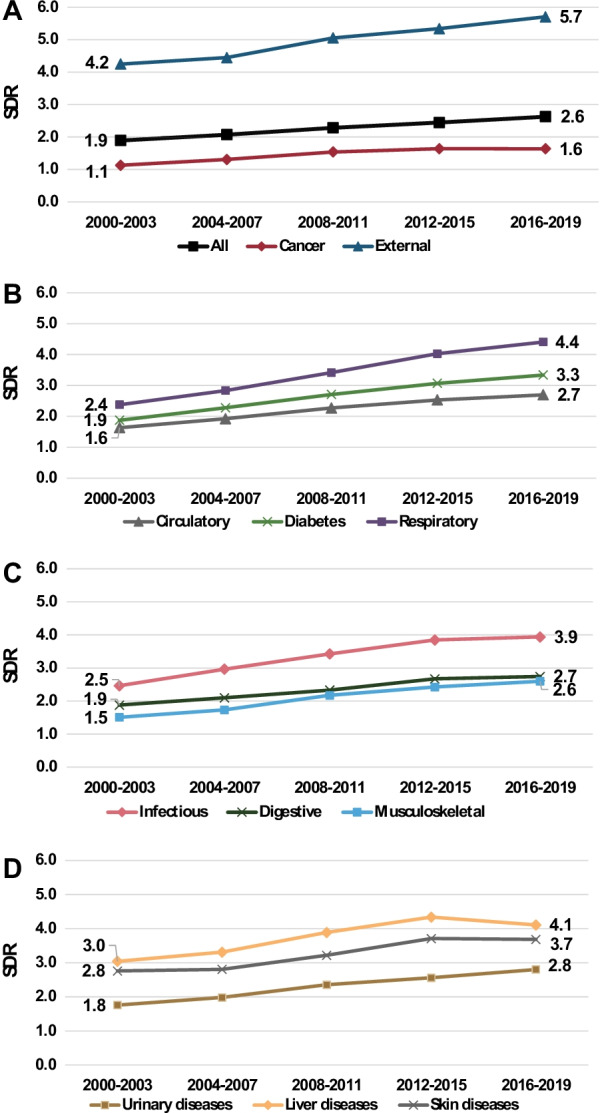
Standardized discharge ratios for acute care discharges from public hospitals, by period and diagnoses, 2000–2019. People with SMI compared to total population, aged 18–74

Figure 4 shows trends in SDRs for total discharges and different diagnostic groups between 2000 and 2019. In general, we see an increasing trend over the period and the ranking of SDRs was maintained throughout. The SDR for total discharges increased by 39% from 1.9 to 2.6, with the lowest SDR for cancer and the highest for external causes (Fig. 4A).

Between 2000–2003 and 2016–2019, the greatest increase was for respiratory diseases and diabetes where the SDR almost doubled from 2.4 and 1.9, to 4.4 and 3.3, respectively. SDRs for circulatory, infectious, musculoskeletal and urinary diseases increased about 60–70% over this period. We note a small decrease in the SDR between 2012–2015 and 2016–2019 for skin and liver diseases while those for cancer and digestive system diseases were stable. The rate of increase of some other SDRs, such as respiratory, infectious and circulatory diseases and diabetes, also appears to be attenuated in this period.

**Fig. 5:**
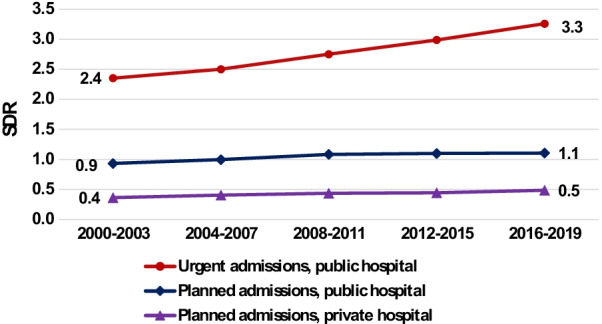
Standardized discharge ratios (SDR) by type of admission and hospital ownership, 2000–2019. People with SMI compared to total population, aged 18–74

SDRs for urgent hospitalizations in public hospitals in Fig. 5 show significantly higher rates among people with SMI, with rising SDRs from 2.4 in 2000–2003 to 3.3 in 2016–2019. However the SDR for planned admissions to public hospitals showed slightly lower rates for the SMI population in 2000–2003, SDR = 0.9, but increased over the study period to 1.1 in 2016–2019. Planned hospitalizations in private hospitals were significantly lower for people with SMI throughout the study period, with a SDR of 0.4 in 2000–2003, increasing to 0.5 in 2016–2019.

Additional file 1: Tables S2 and S3 give the SDRs for Table 1 and Figure 4 for all periods and for Fig. 5, respectively, with 95% confidence intervals.

## Discussion

We found higher morbidity among people with SMI within 5 years of their discharge from inpatient psychiatric facilities, as shown by their acute care hospitalization rates and their diagnoses in public hospitals compared to the total population. Rates of all non-maternity discharges for people with SMI were 2.6 times that of the total population in 2016–2019. This accords with the high prevalence of chronic diseases and risk factors reported in past studies [8–13]. Lurie et al. [20] found a lower excess acute care hospitalization risk of 1.45, but their psychiatric population was derived from community- based health provider diagnoses and included people without psychiatric hospitalizations, and presumably less severe mental conditions than our SMI population.

### SDR by disease group

The highest SDRs were found for external causes, due to the high rate of discharges for suicide and self-injury in the SMI population, reflecting the high rate of hospitalized suicide attempts in those with a background of psychiatric hospitalization. We reported a similar finding in our paper on suicide in people with a past psychiatric hospitalization [25]. We found that while Jews over the age of 17 with a previous psychiatric hospitalization comprised about 2% of the Israeli Jewish population, they accounted for approximately one third of their completed suicides in 2007–2009.

SDRs for respiratory and infectious diseases, particularly for septicemia, were also high. This may be due to higher rates of smoking and drug abuse among the SMI population [9, 12, 13] and high rates of chronic respiratory disease [19]. Higher discharge rates for diabetes and circulatory diseases reflect their higher prevalence in the SMI population, as reported by Levinson et al. [19] and others [12, 13], and can be a result of a tendency to a sedentary lifestyle and unhealthy diet [9] leading to increased rates of metabolic syndrome [10], a strong predictor of cardiovascular disease and diabetes.

Chronic kidney disease has been found to be more prevalent in people with SMI, particularly in those treated with lithium [26], and is also common in people with cardiovascular conditions and diabetes. We found high SDRs for urinary tract diseases, particularly for chronic renal failure (Table 1). The high SDR for digestive system diseases, which include liver diseases, may be due to alcohol abuse and high obesity levels. Poor oral healthcare as reported by de Hert et al. [13] might also contribute to the high SDR for digestive system diseases. Dental caries were similarly a leading diagnosis with higher admissions in the SMI population in the southeast London study [22].

We also found higher levels of discharges for musculoskeletal diseases which may be due to higher osteoporosis [13]. Levinson et al. [19] reported a 2.5-fold risk for chronic pain, possibly due in part to back pain, which could contribute to these discharge rates.

The lowest SDRs were found for cancer. Scott et al. [9] also report that it is not clear whether people with SMI are more likely to develop cancer, although there might be differences between different types of cancer; respiratory cancers are more frequent while breast cancer is less frequent. In Israel, where screening for breast and colon cancers is universal, promoted and funded by the HMOs, the SMI population is likely to be included. Early detection of these common cancers may help prevent later treatments and hospitalizations and maintain a lower differential with the total population.

The SDRs were higher for females than male for almost all diagnoses. Similarly, higher mortality RRs were found for females of all ages compared to males among people with past psychiatric hospitalizations [18] and for people with schizophrenia diagnoses [20], as well as higher standard mortality ratios for suicide rates [25]. Fok et al. also found higher standardized admission ratios for females with personality disorders than for males for most diagnoses [24]. We note that psychiatric hospitalization rates for females are lower than males: administrative data showed higher rates of males hospitalized in psychiatric facilities than females for ages 18 and above, and the age-adjusted rate ratio of male/female psychiatric inpatients was 1.6 in 2020 [18]. Given their lower psychiatric hospitalization rates, it is likely that, in general, women are hospitalized with greater psychiatric disease severity than men. Their higher SDRs than those of males for physical disease may support the suggestion that severity of physical disease is directly associated with severity of mental disease.

Maternity hospitalizations are ‘healthy’ hospitalizations. Our data shows that unlike other acute care hospitalizations, the discharge rate of people with SMI are significantly lower than that expected from the total population and show that females with SMI are less likely to be mothers.

Similar results to ours were found by Jayatilleke et al. [23] in their study of hospitalizations of people with SMI, although their Standardized Admission Rates were lower than our SDRs in 2016–2019. This may be because their study was conducted earlier, in 2009–2010, and used only one diagnosis per hospitalization, while we used all diagnoses. Counting multiple diagnoses per hospitalization will lead to a higher RR given that the less healthy SMI population is likely to have more diagnoses than the total population.

Comparing our results with excess mortality in the SMI population by causes of death in Israel [18] in 2017–2018, we found similarly that the highest excess rates were for suicide (RR = 15.0), other external causes (RR = 4.7), accidents (RR = 3.1), respiratory diseases (RR = 2.4) and diabetes (RR = 2.2), and a lower excess rate for cancer (RR = 1.3). However, the RR for heart and cerebrovascular disease mortality (1.3) was lower than the SDR for circulatory diseases (2.7) found in this study, likely due to our use of all diagnoses. Hypertension is often listed as a secondary diagnosis and is likely to be more prevalent in people with SMI, thus raising the SDR for discharges with a circulatory diagnosis.

### Discharge rate ratios by age and sex

We found lower RRs at the oldest and youngest ages. At ages 65–74, chronic and acute diseases are more prevalent in the total population which appears to offset some of the excess morbidity in the psychiatric population. At ages 18–24, usually a relatively short time after the onset of psychiatric conditions, their effect on physical health may be less pronounced. But even at these ages, the discharge rates of those with SMI from acute care hospitalizations were more than twice those expected from the total population rates for most diagnostic groups. In older adults, the RRs peaked at much higher values, which may be due to earlier and more severe onset of physical disease in the SMI population. Diabetes seems to be an exception, with very high RRs at younger ages, which may reflect a strong predisposition to diabetes in younger people with SMI, while at older ages, the general population is also susceptible to developing this disease.

The total discharge RR is lower in young women aged 18–34 than in young men (Fig. 2) because at child-bearing ages a large proportion of non-maternity hospitalizations are for women being treated for perinatal and gynecological conditions [27]. Ministry of Health administrative data for the total population shows that for these age groups women's hospitalization rates were 1.5–2.5 times those of men [27]. As we saw from the low SDR for maternity hospitalizations, women with SMI are less likely to give birth, and therefore their share of perinatal and gynecological hospitalizations is also lower, leading to lower SDRs than for men at these ages.

### Trends in SDR over time

While acute care hospital discharge rates decreased between 2000 and 2019 for the total population, they increased for the SMI group (Fig. 1). Hence the ratio of the SMI rates to the total population rates, the SDR, increased for total discharges and for all reported diagnoses. This is to be expected in view of the reforms in the mental health system in Israel. Following the reduction in psychiatric hospital beds and increasing use of community-based rehabilitation we have seen falling rates of psychiatric bed-days/year, and of people hospitalized, in particular for those hospitalized for a year or more [18]. With the increase in community-based treatment, it is likely that those hospitalized in recent years have more severe mental disease than those hospitalized in previous years, which would influence our study population with SMI, defined as those who had a psychiatric hospitalization within the 5 years preceding the acute care hospitalization. We conjecture that the physical comorbidity of people with SMI increases directly with the severity of their mental disease, and therefore they have higher rates of acute care hospital discharges compared to the total population, causing the increase in SDRs.

A similar increasing trend can be seen in the OECD data for Israel for excess mortality in people with schizophrenia and bipolar disease [6], calculated for ages 18–74. However, the rate ratio of age-adjusted mortality rates for people with SMI of all ages compared to the total population has remained stable [18], probably since it is lower in the elderly aged 75 and over, the age of most deaths.

We noted a small decrease or stable values of SDR for some diagnoses between 2012–2015 and 2016–2019—digestive system (including liver), musculoskeletal and skin diseases and cancer. This is hopefully a result of the reform uniting the responsibility for mental and physical health under the HMOs, but it is still too early to draw definitive conclusions. These measures need to be tracked further to see whether the positive trends continue.

### Hospitalizations by admission type and hospital ownership

Urgent hospitalizations in public hospitals had much higher rates for the SMI group, but planned hospitalizations rates in these hospitals were similar or only slightly higher than the general population over the study period. SDRs for planned hospitalizations in private hospitals showed that rates for people with SMI were much lower, about half those of the general population, although in both private and public hospitals the SDRs for planned hospitalizations increased slightly over the years (Fig. 5, Additional file 1: Table S3). Given the higher morbidity in the SMI population, it is particularly important for them to have consistent preventative care and follow-up through planned treatments and operations, to heal and prevent deterioration in their physical condition. Hopefully, the increase in planned treatments will continue.

The lesser use of private hospitals by the SMI population is to be expected as their mental disease generally leads to lower economic status. For example, Davidson et al. found that only a small percentage of people in Israel who had been hospitalized with schizophrenia/acute psychotic disorders or with bipolar disease (10–25%) were employed and earning minimum wage or above [28]. Therefore, it is harder for them to afford treatment in private hospitals. However, the SDR in private hospitals of 0.4–0.5 which shows that some do receive treatment there, albeit less than the total population, may be since the cost is partially covered by inexpensive supplementary medical insurance.

### Health policy implications and recommendations

We showed here, as have other studies, that people suffering from SMI are at an increased risk for physical illness, both due to the influence of their mental health on their ability to care for themselves and due to possible side effects of their medications. For many years, the responsibility for mental and physical health was divided. Since the mental health insurance reform, the responsibility for both aspects rests with the HMOs and this is an opportunity for improved physical health care, the importance of which cannot be overemphasized. It is essential to regularly monitor disease risk factors such as blood pressure, blood sugar and lipids. People with SMI need to be encouraged to live a healthier lifestyle, stop smoking, do more physical activity, lose weight and eat better diets.

All professionals involved in the care of the SMI patients, including their family doctor, the psychiatric hospital and community care team, and rehabilitation coordinators, need to be aware of these goals, and pool and collaborate their efforts to promote physical health care, too. This is not yet the case as, for example, a recent presentation which reported finding that psychiatrists treating SMI patients showed less encouragement and fewer referrals to smoking cessation programs than general practitioners [29]. A similar study found less SMI patients complete such programs compared to those without SMI [30].

Morden et al. have discussed the role of the family physician in the USA in the care of patients with SMI, and present strategies for providing care in collaboration with psychiatrists [31]. In Israel, we are at an advantage compared to the USA, since our socialized health system reduces financial barriers to care and cooperation, and our electronic record system allows easy sharing of clinical data. Other suggestions they make, such as developing residency curricula on collaborative mental health care and studying methods for successful behavior modification in people with SMI, would be valuable in Israel.

The national program for health quality indicators in the community added a set of mental health indicators in 2015, which include indicators for continuation of care for those hospitalized, diabetes prevalence and follow-up and BMI prevalence [32]. The addition of further indicators for the maintenance of health in this population might be an incentive for improvement.

The National Councils for Health, a set of advisory bodies to the Ministry of Health, have recently formed an inter-council committee to assess and discuss ways for improving the physical health of people with SMI, and provide recommendations. In a summary of their meetings [33] they report on the efforts of each of the HMOs, and the rehabilitation services, to include physical health promotion in their mental health treatment programs. More needs to be done, and their recommendations include better integration of physical and mental healthcare and improving prevention, screening, treatment and follow-up of physical diseases. Financial incentives are needed to encourage the HMOs and the practitioners to invest in these goals, with a possible Ministry of Health program to organize this.

We have seen a small improvement in the trends of excess hospitalization of people with SMI compared to the total population, possibly due to the insurance reform, and we hope this will continue and progress.

Community care as an alternative to hospitalization for the SMI population must be readily available. It is important that the creation of a national database on community treatments for the SMI population, currently in progress, be completed including data from all the HMOs, to enable monitoring the availability and trends in provision of care.

### Strengths and limitations

The strength of this study is the large nationwide databases available for two decades, allowing assessment of trends and disease in smaller diagnostic groups.

A limitation of this study is that we do not have individual level data on risk factors for disease such as smoking, BMI and lipid levels, or socio-economic data such as education level, which could be controlled for in regression models.

We also do not have comparative data from the period before the mental health system reforms, since the National Hospital Discharges Database (NHDD) was not complete then. In addition, there were major population changes in the 1990s with the large immigration waves from the former Soviet Union and Ethiopia, which would be hard to control for in our analysis.

## Conclusions

This study has shown higher acute-care hospitalization rates in people with recent psychiatric hospitalization for SMI compared to the total population, with the largest gap for respiratory, infectious and skin diseases and diabetes. The differences increased between 2000 and 2019, possibly because psychiatric hospitalization in recent years has been for those with more severe mental disease than in previous years, as less severe cases are likely to be referred to community treatment. We recommend that mental health and general health professionals collaborate to ensure good physical healthcare, health promotion and disease prevention in the SMI population.

## Supplementary Information

**Additional file 1: Table S1**. Rate ratios of discharges of SMI group compared to total population, 2016–2019, with 95% CI, by age and diagnoses, and by sex for total discharges. **Table S2**. Standardized discharge ratios (SDR) for SMI group compared to total population, aged 18–74, with 95% CI, by period of discharge, sex and diagnoses. **Table S3**. Standardized discharge ratios (SDR) for SMI group compared to total population, aged 18–74, with 95% CI, by period of discharge, type of admission and hospital ownership.

## Data Availability

Due to restrictions of privacy on the data it is not available to the public, although the Ministry of Health periodically publishes summary data, and will supply such data on request.

## Abbreviations

AOR: Adjusted odds ratio
HMO: Health Maintenance Organization
NHDD: National Hospital Discharges Database
NPCR: National Psychiatric Case Registry
RR: Rate ratio
SDR: Standardized discharge ratio
SMI: Serious Mental Illness
